# Validation of scoring system for preoperative stratification of intra-operative risks of complications during cataract surgery: Indian multi-centric study

**DOI:** 10.4103/0301-4738.49396

**Published:** 2009

**Authors:** Vinay Agrawal, Jinish Upadhyay, Indian Cataract Risk Stratification Study group

**Affiliations:** Pramukhswami Eye Hospital, Chunabhatti Station Road, Chunabhatti, Mumbai, India

**Keywords:** Cataract surgery, intraoperative, risk stratification

## Abstract

**Aim:**

To validate a system that uniformly and objectively assesses the risk of complications of cataract surgery performed with phacoemulsification technique in individual patients preoperatively.

**Materials and Methods:**

Outcome analysis of patient data entered into a standardized protocol. The data sheet was analyzed at a single center in terms of the risk assessed preoperatively and the incidence of surgical complications. This study did not assess the final visual outcome of eyes with complications. Each patient was categorized into a risk group according to the number of points scored. Group 1 (no added risk) 0 points, Group 2 (low risk) 1–2 points, Group 3 (moderate risk) 3–5 points, Group 4 (high risk) 6 points or more.

**Results:**

The number of eyes in each risk group was 2894 in Group 1 (44.1%), 1881 in Group 2 (28.6%), 1575 in Group 3 (23.9%), and 214 in Group 4 (3.3%). A total of 6564 eyes were assessed, of these 3669 eyes (55.9%) had a minimum of one risk factor and were thus not “routine”. The group-specific events of complications were Group 1, 46 (1.6%), Group 2, 108 (5.7%), Group 3, 168 (10.7%), and Group 4, 69 (32.2%). The total incidence of complications was 5.7%. The group-specific rate of intraoperative complications increased through the risk groups (*P* < 0.001).

**Conclusion:**

The study validates a scoring system that is predictive of intraoperative complications. This system uses information that is readily available from the preoperative history and assessment of the patient.

Various risk factors impact the outcome of cataract surgery. Each of these risk factors increases the possibility of an adverse outcome during cataract surgery. However, until recently, prediction of the likelihood of a complication during phacoemulsification surgery has been based on a “subjective” assessment of the patient by the surgeon. Various studies have identified individual risk factors that increase the risk of intraoperative complication.[1–5] The analysis from these suggests that not all cataract surgeries present the same degree of complexity. Risk stratification, is acceptance of the fact that not all cases are the same and some cases will be more prone to develop complications because of the technical difficulty and/or structural weakness of tissues.[6]

There are a number of reasons to attempt risk stratification in cataract surgery:

1. This will allow meaningful preoperative counseling of patients.
2. This will allow teaching centers to assign low-risk cases to the novice surgeons, and higher risk cases can be assigned to advanced trained surgeons.
3. Each surgeon can then assess his case results against a standard benchmark. This will allow review of steps needed to conform to the benchmark, by either seeking more training or referring more complex cases to higher centers.
4. This permits meaningful comparison of data between individual surgeons and hospitals with differing case mix.

Muhtaseb *et al.*,[7] have developed a system of patient classification to uniformly and objectively assess the risk of complications in individual patients preoperatively. The present multicenter study was conducted in nine centers across India. The aim of the study was to confirm the broad validity of the cataract risk stratification system proposed by Muhtaseb *et al.*[7]

## Materials and Methods

Nine sites participated in the data collection phase. Each operating surgeon who also personally allotted the points preoperatively was a consultant grade surgeon with more than five years of surgical experience in phacoemulsification. Data was submitted to the central database on a monthly basis. The study was carried out over a period of 15 months from March 2005 to May 2006.

A data sheet was created to include the patient characteristics to be used in the scoring protocol [Table 1]. Points were allotted to each risk factor according to its potential for increasing surgical risk.

**Table 1:** Patient characteristics used in the scoring protocol

Category 1	Category 2 (1 point each)	Category 3 (3 points each)
No additional risk factors	Previous vitrectomy	Dense/total/ brunescent/black/ white cataract
	Corneal scarring	Pseudoexfoliation
	Small pupil (<3 mm)	Phacodonesis
	Shallow anterior chamber (depth <2.4 mm)	
	Age >75 years	
	High ametropia (>6D myopia of hypermetropia)	
	Posterior capsule plaque	

Risk assessed by surgeon D - Diopter

The data sheet was attached to the case notes and in the preoperative assessment the surgeon would indicate the presence of any risk factors. Each patient was categorized into a risk group according to the number of points scored. Group 1 (no added risk) 0 points, Group 2 (low risk) 1–2 points, Group 3 (moderate risk) 3–5 points, Group 4 (high risk) 6 points or more. The surgeon decided the threshold for transition between each group empirically.

The mode of anesthesia during the surgery varied according to surgeon preference and included topical, subconjunctival, and peribulbar anesthesia. All the patients had an intraocular lens (IOL) inserted in-the-bag unless the event of a complication necessitated the placement into the sulcus or the anterior chamber.

Once the operation was completed the data sheet was used to record the date of surgery, right eye or left eye, and whether a complication had occurred. In the event of a complication having occurred, its nature was specified as follows: incomplete capsulorrhexis, posterior capsule tear, vitreous loss, zonule dehiscence, lost nucleus, anterior capsule tear, unplanned extra capsular extraction (ECCE), corneal burn, wound leak. An option to mark other was provided. In this case the surgeon could specify the complication.

The data so collected was entered into a computerized datasheet (Microsoft Excel 2003) and subjected to statistical analysis. We used the χ^2^ test (or Fisher's exact test for small data sets) for statistical analysis. The data collection for the study lasted for 14 months. A total of 6,564 eyes of 6564 patients were included in the analysis.

## Results

We analyzed data on 6564 patients included in this study. Surgery was performed on 3294 males (mean age 60.3 ± 11.84 years) and 3270 females (63.4 ± 12.34 years). There were 3363 right and 3201 left eyes. The number of eyes in each risk group was 2894 in Group 1 (44.08%), 1881 in Group 2 (28.65%), 1575 in Group 3 (23.99%), and 214 in Group 4 (3.26%). Thus 55.9% of patients had a minimum of one risk factor and were thus not “routine”.

The group-specific events of complications were Group 1, 46 (1.58%), Group 2, 108 (5.74%), Group 3, 168 (10.66%), and Group 4, 69 (32.24%). The total incidence of complications was (5.66%). The group-specific rate of intraoperative complications increased through the risk groups (*P* < 0.001). There was a strong statistical significance in the complication rate between the different risk groups. This shows that the risk of intraoperative complications rises with every group which needs to be taken into account by the surgeon. The risks of complications that show an increase through the categories are summarized in Table 2. Of all the complications, anterior capsular tear, Descemet's membrane detachment and unplanned ECCE did not reach statistical significance.

**Table 2:** Complication rates through risk groups

	Group I (0 points) n=2894 (%)	Group II (1– 2 points) n=1881 (%)	Group III (3–5 points) n=1575 (%)	Group IV (6 points) n=214 (%)
Incomplete capsulorhexis	9 (0.31)	12 (0.64)	11 (0.69)	18 (8.40)
PC tear	11 (0.38)	43 (2.28)	32 (2.03)	16 (7.47)
Vitreous Loss	17 (0.58)	34 (1.80)	46 (2.92)	14 (7.47)
Zonular Dehiscence	3 (0.10)	4 (0.21)	9 (0.57)	3 (1.40)
Nucleus drop	2 (0.07)	7 (0.37)	13 (0.82)	9 (4.20)
Anterior capsular tear	0 (0)	0 (0)	25 (1.58)	0 (0)
unplanned ECCE	0 (0)	4 (0.21)	15 (0.95)	3 (1.40)
Corneal Burns	0 (0)	4 (0.21)	15 (0.82)	6 (2.80)
Descemets tear	4 (0.14)	0 (0)	2 (1.58)	0 (0)
Total	46 (1.59)	108 (5.74)	168 (10.66)	69 (32.24)

PC - Posterior capsule, ECCE - extracapsular cataract extraction

## Discussion

Our results support the validity of the scoring protocol of Muhtaseb *et al.*[7] The advantage of this system is that it is simple and easy to apply in the clinical setting. This will be important for its application on a wider scale. The scoring system has shown itself to be predictive of intraoperative complications using information that is readily available from the preoperative history and assessment of the patient. However, as noted by Muhtaseb *et al.*,[7] there was no scheme for weightage of the reviewed papers to determine their influence on the system. It is also important to note that our study defines old age as 75 years. This is different from the Western literature where old age is defined as greater than 88 years. This is because the average life expectancy in our country is lower than the Western countries.

The present trial follows the methodology of Muhtaseb *et al.*[7] Thus it suffers the potential shortcomings of the same. For example, there are no points allotted to traumatic cataract. This is a situation that regardless of zonular trauma can lead to a significantly increased risk of posterior capsular rupture. There is no consideration given to the incidence of complication during surgery in the other eye. This has been shown to increase the risk of complication in the fellow eye.[8] However, despite these shortcomings it puts into place a system that will allow preoperative scoring of the risk factors. It was mentioned in the original work that the open nature of the system could lead to a skewed recording of risk. However, we did not find any misuse of the miscellaneous risk option in the study.

In our opinion the use of this system will allow appropriate case selection for trainee surgeons. This would effectively tailor the cases to each surgeon based on the trainee's surgical expertise. Though it has been argued that this can limit the trainee's experience to only “simple” cases,[9] we feel that this system should be used to track each surgeon's increasing experience to permit a more complicated case mix for surgery in a controlled manner. This can be further improved by validating the risk of complications in the hands of a trainee over a period of time. This will allow a better assessment of cataract surgical complications and probably help in improving future grading systems.[10]

The use of this system will allow surgeons to obtain accurate informed consent from patients. The patients in the higher risk group can thus be informed of the higher possibility of complications and the outcome resulting from it. However, it should be kept in mind that there are other possible outcomes like suprachoroidal hemorrhage, wound leak, IOL mispositioning that can occur across risk groups.

This system can also be used by hospitals to accurately assess their outcomes depending on the case mix seen by them. This will allow comparison between different centers in a meaningful manner. The data submission from the 12 centers has not been presented individually as this was not the aim of the study. However, a trend towards poorer overall outcomes was noticed in two centers that had a higher mix of Grade III and IV surgeries. This indirectly validated the basis of this study. This system can also be used to set up a national outcome registry to enable further consolidation of quality care in the national blindness control programs across the country. This will definitely improve the standard of care in a country where poorly performed cataract surgery is second only to cataract as a leading cause of blindness.[11]

This study helps us to understand the need to segregate cataracts according to the risk inherent in performing the surgery. It also allows us to put into perspective why some cases do better than others. An adaptation of guidelines based on such risk stratification studies in future, will allow us to achieve safer surgery for our patients and a better understanding of the risks by the patients.
